# ‘I’m fine!’: Assertions of lack of support need among patients with chronic obstructive pulmonary disease: A mixed-methods study

**DOI:** 10.1177/17423953211000386

**Published:** 2021-03-15

**Authors:** A Carole Gardener, Caroline Moore, Morag Farquhar, Gail Ewing, Efthalia Massou, Robbie Duschinsky

**Affiliations:** 1 Department of Public Health and Primary Care, Forvie Site, Cambridge Biomedical Campus, Cambridge, UK; 2 School of Health, Wellbeing and Social Care (HWSC), Faculty of Well-being, Education and Language Studies, The Open University, Walton Hall, Milton Keynes, UK; 3 School of Health Sciences, University of East Anglia, Norwich, UK; 4 Centre for Family Research, University of Cambridge, Cambridge, UK

**Keywords:** Chronic Obstructive Pulmonary Disease, support needs, compliance, help-seeking, disavowal

## Abstract

**Objectives:**

To understand how people with Chronic Obstructive Pulmonary Disease (COPD) disavow their support needs and the impact on care.

**Methods:**

Two stage mixed-method design. Stage 1 involved sub-analyses of data from a mixed-method population-based longitudinal study exploring the needs of patients with advanced COPD. Using adapted criteria from mental health research, we identified 21 patients who disavowed their needs from the 235 patient cohort. Qualitative interview transcripts and self-report measures were analysed to compare these patients with the remaining cohort. In stage 2 focus groups (n = 2) with primary healthcare practitioners (n = 9) explored the implications of Stage 1 findings.

**Results:**

Patients who disavowed their support needs described non-compliance with symptom management and avoidance of future care planning (qualitative data). Analysis of self-report measures of mental and physical health found this group reported fewer needs than the remaining sample yet wanted more GP contact. The link between risk factors and healthcare professional involvement present in the rest of the sample was missing for these patients. Focus group data suggested practitioners found these patients challenging.

**Discussion:**

This study identified patients with COPD who disavow their support needs, but who also desire more GP contact. GPs report finding these patients challenging to engage.

## Introduction

Patients with chronic conditions can be reluctant to say they need help and support. Identifying need is not always comfortable or desirable for patients and may not always feel legitimate to them.^1^ A wide literature^2–7^ exists in medical sociology on the ways individuals resist being conscripted to the sick role and assert their lack of support needs. One component of this is verbal disavowals of support needs. In a study of 2,000 adults commissioned by the Mental Health Foundation^8^ almost a third of those surveyed said they often lied about how they were feeling to other people. The Mental Health Foundation claimed that the average adult will say ‘I’m fine’ fourteen times a week, only 19% of the time accurately representing their sense of wellbeing. Dozier and colleagues^9^ found that mental health patients who disavow emotional needs in interviews with clinical researchers are at greater risk of treatment non-adherence, and their carers report higher levels of depression. Disavowal of emotional needs in clinical interview was operationalised by these researchers on the basis of discursive markers of a speaker’s implicit recognition of emotional needs and desire for support combined with explicit assertions that no needs are present and no support is needed.^10^ Subsequent work by Caspers and colleagues has replicated the finding of lower rates of treatment participation among patients using supportive services for drug dependence.^11^ Yet Fonagy and colleagues have found that, among those who do engage with supportive services, they may see the greatest improvements in functioning.^12^ To date, however, the disavowal of need has not been explored in patients with a chronic physical health condition.

An exemplar physical condition is advanced chronic obstructive pulmonary disease (COPD). This condition is sometimes associated with stigma and shame, due to its association with smoking and perception as self-inflicted. There has been work to date on patient negative self-perceptions^13,14^ and one study has examined how patients with COPD may attribute symptoms to age.^15^ However, as yet, there has been little focus on patient disavowal of support needs, and the wider implications of this for patients, outcomes or the provision of healthcare. This is important since patient disavowal of support needs may result in under- or mis-utilisation of services, something known to be relevant to patients with COPD where patients report a range of support needs.^16,17^ This study therefore sought to understand how people with COPD disavow their support needs and how this influences their care. To do this we aimed to (i) explore other facets of narratives provided to researchers as part of a mixed-method study in which there were indicators of disavowal of needs; (ii) examine correlates in survey data of patient disavowals relevant to the patient’s mental and physical health and service needs; (iii) consider implications for clinical practice with such patients in primary care settings.

## Methods

### Study design

The design was a mixed-method study comprising two stages:

Stage 1: Sub-analysis of existing data collected within the Living with Breathlessness study program^18^ involving:

- identification of cases of disavowal of support needs
- qualitative analysis of patient interviews
- analysis of linked quantitative questionnaire data

Stage 2: Focus groups with healthcare practitioners in primary care

Ethical approval for the Living with Breathlessness Study programme was obtained from the National Research Ethics Service Committee East of England – Cambridge South (Reference number 12/EE/0163). Ethical approval for additional health care professional focus groups was obtained from the University of Cambridge Psychology Research Committee, Application No.PRE.2017.039.

### Context

The Living with Breathlessness (LwB) study^18^ was a prospective mixed-method multiple-perspective longitudinal programme of work that sought to improve care and support for patients and informal carers living with advanced COPD

Stage 1 of the current study described below involves sub-analysis of data from a component of the LwB study, [the LwB-Longitudinal Interview Study (LwB-LIS)]. The LwB-LIS comprised a 18 month follow-up study of 235 patients with advanced COPD and their informal carers (n = 115), involving 3-monthly mixed-method semi-structured interviews using flexible methodology to capture changing function, support needs and service-access. Participants in the LwB-LIS were recruited from primary care sites across the East of England (see Table 1 for LwB-LIS study characteristics) and comprised a population-based sample of 235 patients including 143 men and 92 women aged from 36 to 92 years old, with a mean age of 71.6 years. Data collected from within the study included qualitative interviews covering life with advanced COPD, experience of symptoms, medication and contact with health care professionals (HCPs), and informal support. Quantitative data were collected firstly via patient self-report in response to questions addressing demographics, health status, healthcare usage, current medication and care needs. In addition, patients completed three self-report questionnaires widely used in clinical practice with this patient group: the Chronic Respiratory Questionnaire (CRQ),^19^ the COPD Assessment Test (CAT)^20^ and the Hospital Depression and Anxiety Scale (HADS).^21^ The CRQ measures quality of life in chronic lung disease: the 20-question self-report version (CRQ-SR) covers dyspnoea, fatigue, emotional functioning and mastery which form two subscales for physical and emotional functioning (CRQ–Emotional and CRQ–Physical). The CAT (eight questions) assesses COPD impact, for example, shortness of breath and ease of living at home. The HADS (14 questions) consist of two subscales to screen for anxiety (HADS-A) and depression (HADS-D).

**Table 1.** LwB study characteristics and inclusion and exclusion criteria.

LwB’s longitudinal interview study (LIS) characteristics	LwB LIS inclusion criteria	LwB LIS exclusion criteria
Population-based longitudinal mixed-method cohort study Recruitment via East of England primary care practices Recruited: 235 patients Audio-recorded mixed-methods interviews (in participants’ location of choice) Quantitative data: demographics, co-morbidities and service use and disease specific health-related quality of life and psychological health using validated questionnaires Qualitative data: living with advanced COPD, self-identified need, views on formal and informal care and thoughts on future care	Patients with COPD meeting two or more of the following: FEV1 < 30% 2+ exacerbations requiring prednisolone and antibiotics in the previous year Long-term oxygen therapy Cor pulmonale MRC dyspnoea scale 4+Admission for COPD in previous year	Patients with any of the following: Serious mental health problemSerious learning difficulty Active cancer Active alcoholism

#### Stage 1: Sub-analysis of existing data collected within the LwB-LIS study

Stage one involved three steps: 1) identification of cases of disavowel of needs; 2) analysis of qualitative interview data and 3) quantitative analysis of linked data.

##### Identification of cases of disavowal of support needs

LwB-LIS baseline interview and questionnaire data were purposively sampled for caseness (of disavowal of needs) of patients living with advanced COPD.

In order to identify potential cases of disavowal of needs a set of criteria, used in previous mental health research since Dozier and colleagues,^9^ was minimally adapted for applicability to an interview with COPD patients (see Table 2). This adaptation was supported by piloting on a set of eight transcripts. The criteria included evidence of the participant minimising the effects of COPD on their lives, lack of memory when asked about negative experiences, and characterising the self as not warranting resources or support.

**Table 2.** Criteria used to identify ‘I’m fine’ patient cases.

Criteria	Example quote
Minimising the effects and symptoms of COPD	*“So I have that sort of experience (breathlessness) but nothing that’s going to create problems for me if you know what I mean? Just annoying, irritating.”* (203–410)
Normalisation	*“I don’t think I walk slower than people the same age”* (610–005)
Contradiction between symptom indicators and significance	*“… I mean, the breathlessness, as you can tell, it doesn’t bother me at the moment, but if I go out walking, it’s difficult going up hills…but other than that…I walk quite well. I could run if I wanted to.”* (016–302)
Language use (stoicism, lack or flattening of feeling words) to downplay needs	*“But I mean I’m OK. I make myself OK. It’s why I don’t trouble the doctors. If I feel bad I know what I have to do…so you just get on with it, it’s a fact of life.”* (010–301)
Symptom comparison to downplay self’s needs	*“But when you are breathless you’re not breathing [pants} See I’m not like that…I mean emphysema. I mean my bother in law…he’s..he’s got emphysema. I mean I don’t think I’ve got it*” (005–200)
Not warranting resources or support	*“… I still feel this thing I don’t want anyone to come in and do something that I can do.”* (017–205)
Lack of memory of negative experiences	*“Well, I can’t remember really [how long I have had COPD] because I’m quite active. How long ago is it since? I’m still active*.” (202–610)
Focus on downplaying own health needs	*“…I’ve gone down with this chest infection […]’ So I do need to do something about that in the New Year.”* (017–205)
**Number of identified cases**	**21 patient cases**

Transcripts of baseline interviews with patients (n = 235) were reviewed by CM to identify potential instances of disavowal of needs (herein referred to as “cases”) based on the criteria. Three members of the study team then independently reviewed the identified candidate cases (n = 31). Discrepancies in the selection process were resolved by discussion. Caseness was established by identifying the extent to which a case met the criteria (cases met on average 5 of the criteria), and all three members of the team agreeing that these represented valid examples. In total twenty-one patients were identified as cases based on the criteria. There were no significant differences between these patients and the rest of the sample (non-cases) in terms of distribution by age, gender or the number of self-reported comorbidities. Differences were assessed by using chi square test for categorical variables and t-test for continuous variables.

#####  Analysis of qualitative interview data

Interview transcripts for both I’m Fine cases (n = 21) and non-cases (n = 10) were analysed to examine differences in the way the ‘I’m Fine’ cases expressed and managed their needs in comparison to the non-cases. To identify a sample of non-cases, random sampling was used: this addressed difficulties in case-matching due to a lack of sufficient certainty about the expected covariates for disavowal or acknowledgement of needs. Data were analysed using conventional content analysis.^22^ Transcripts were read and re-read independently by two team members (CM and CG) in order to identify emerging categories. Categories were discussed and compared by the wider study team, enabling clarification and agreement in regard to interpretation. Case and non-case transcripts were compared for differences in occurrence of categories across the two patient groups. Only categories that were not directly linked to the sampling criteria were used to identify disavowal of needs. After analysis of 10 non-cases, no new information was forthcoming, indicating data saturation had been reached.

##### Analysis of linked quantitative questionnaire data

As noted above, the LwB-LIS collected patient data from a wide range of quantitative measures. These were reviewed to identify variables potentially relevant to patient disavowal of patient’s mental and physical health and service needs. Identified variables included: clinical contacts; desired clinical contacts; number of exacerbations requiring/not requiring the use of antibiotics, and patient scores on the mastery domain of the CRQ,^19^ the CAT^20^ and the HADS.^21^ All data related to patient experiences over the previous three months.

Comparison of data from cases and non-cases was undertaken using chi square tests for categorical variables and t-tests for continuous variables. Categorical data were reported as percentages. Descriptive statistics were reported for continuous scores, specifically the mean value and standard deviation. In the latter case assumption of normality was tested with Q-Q plots, and assumption of homogeneity of variance using Levene's test of equality of variances. In cases of unequal variances, the corresponding result of the t-test was used to correct for the lack of homogeneity. In addition, the effect of risk factors relating to HCP involvement (age, anxiety and depression [HADS scores], and the impact of COPD on daily life [CAT scores]) was examined using a series of logistic regression models. In this case HCP involvement was identified by patients being able to identify an HCP who had given them support in the last three months.

Significance level for all analyses was 0.05. All analyses used SPSS 27.

#### Stage 2: Focus groups with healthcare practitioners in primary care

Focus groups involving HCPs who worked with patients with COPD were conducted to explore the relevance of the Stage 1 findings to current clinical practice.

##### HCP recruitment

Two primary care practices in the East of England, recruited via the Clinical Research Network, identified HCPs within their practice who worked with patients with COPD. Eligible HCPs were posted recruitment packs containing an invitation letter, participant information sheet, reply form and pre-paid envelope. HCPs interested in participating returned completed reply forms to the study researcher who then contacted them to arrange the focus groups.

Nine HCPs agreed to participate in the focus groups (45% response rate based on number of packs distributed within the practices). Five of the participants were GPs, three were practice nurses and one was an assistant practitioner. The number of years they had worked with COPD patients ranged from five to 30 years.

##### Data collection

Two focus groups were conducted in January 2018. In order to support attendance groups were run in the HCPs’ place of work. Written informed consent was obtained from all participants. Participants were provided with lunch and completed a brief demographic questionnaire. Using a topic guide, informed by the Stage 1 results, participants were asked to discuss a range of areas including: 1) whether they recognised patients with the ‘I’m Fine’ profile and 2) ways of improving support for this patient group (See Table 3). Each group was facilitated by CM, lasted approximately 45 minutes, and was audio-recorded with the participants’ permission.

**Table 3.** HCP focus group topic guide: ‘I’m fine’ study.

1. Topics to cover	Self-management attitudes and beliefs regarding COPD Attitudes and beliefs of ‘I’m fine’ patients towards health services and HCPs
2. Questions	Have you ever come across patients that fit this profile in your practice? What are your initial thoughts about ‘I’m fine’ patients? What do you think are the implications of this for practice? How do you think these issues could be addressed?How could you help these patients to think about their support needs? If patients feel reluctant to seek healthcare advice how do you think you could help them to identify and address their problems? What is your experience of COPD annual reviews and how could they be used to identify ‘I’m Fine’ patients and their support needs?

##### Data analysis

Audio recordings were fully transcribed, checked for accuracy and anonymised. Data were again analysed using conventional content analysis. As in Stage 1 transcripts were read and re-read independently by two team members (CM and CG) in order to identify emerging categories. Each potential category was discussed, compared and agreed within the wider study team.

## Results

The following section outlines the results from the Stage 1 and Stage 2 analysis. The Stage 1 results report on findings from the comparison of ‘I’m Fine’ cases and non-cases, explored via the sub-analysis of data from the LwB-LIS study. These are presented in two sections:1) the analysis of qualitative interview data and 2) the analysis of linked quantitative questionnaire data. The Stage 2 results summarise key findings from the HCP focus groups.

### Stage 1: Sub-analysis of existing data collected within the LwB-LIS study

####  Analysis of qualitative interview data

Two characteristics were identified which distinguished cases from non-cases, beyond meeting the criteria indicating disavowal of needs. These were: i) non-compliance with symptom management strategies and ii) avoidance of planning for the future.

##### Non-compliance with symptom management strategies

All patients reported being prescribed and taking a range of medications (e.g. inhalers, antibiotics and steroids) as well as engaging with a range of other symptom management strategies including use of oxygen, smoking cessation, and diet and lifestyle changes. Many were also taking medication for a range of co-morbidities. Overall patients accepted these as part of life with a long-term condition and many outlined benefits they gained from different aspects of their management regime.

However, in contrast to non-cases, patients disavowing needs expressed elements of non-compliance with aspects of their management plan, including holding off using antibiotics when they had a chest infection or resisting use of oxygen or anti-depressants. For some this was linked to a belief that they could manage some symptoms using their own resources:

I must say when I had my pneumonia they said ‘use that [inhaler] four times a day,’ but I never bother. […] Because when I walk to the (shopping centre) I’m out of breath because of my walk…. So it’s not a reason, it seems to me, why I should take it. What I need is to rest and then I’ll recover, and I think it’s just not necessary to take it. [P 023-41}

Alternatively, others who disavowed their needs, frequently made assertions of the futility of taking medication:

I haven't told them in the surgery. They're not that bothered about it anyway. But 11 days ago, I decided that the [second inhaler] was making me cough – just a tickle. And it wasn't that there's something wrong, more wrong, with my lungs, it was every time I used it. And I thought “what would happen if I stopped everything, and just used this?” And truthfully, there was no difference whatsoever …. [P 702-100]

##### Avoiding planning for the future

Within the LwB-LIS study, patients were asked explicitly whether they had thought about their future care needs. In contrast to non-cases, few of those who disavowed needs (cases) mentioned future care and, where they did, discussion was only in the most general and distancing of terms (‘*we are all going to die*’) or in relation to co-morbid conditions rather than their COPD:

Well, the care I need in future I feel might not necessarily be anything to do with my lungs. As long as I get the medication, my lungs don’t really worry me. But I do have anxiety about the deterioration in my limbs and my hands and so on, because that obviously is going to have an effect. [P 675-710]

Notably, the majority of cases stated explicitly that they had not considered future care needs. These patients justified avoidance of future planning to avoid negative emotions:

Otherwise you start worrying again don't you? ……I’ve lost five mates this year ….so you know …they are all dead. Cancer. [P 620-300]

Or they expressed the futility of thinking about, and planning for, the future, noting both the unknown quantities involved and the inevitability of having to deal with these issues at a later date anyway. This justified a more fatalistic approach: *“what happens will happen anyway and I’ll deal with it when it comes.”* [P 004–510]

It is also noteworthy that none of the patients who disavowed needs in interviews reported engaging in any planning for their future care with their family, friends or HCPs. The future involvement of family, or enhanced health and social care, was sketched in very general terms where it was addressed at all. This was in contrast to the non-cases who frequently described having discussed the future with others and developed specific ideas about the sort of care and support they would like.

#### Analysis of linked quantitative questionnaire data

Key correlates from the self-report measures between cases (people who disavowed needs) and non-cases are outlined below, together with the effect of risk factors in relation to HCP involvement.

##### Comparison of patient self-report measures

The assumption of normality was met for both HADS scores. HADS anxiety scores had unequal variances (F = 4.70, p = 0.031) between cases and non-cases. The mean anxiety score on the HADS for patients who disavowed needs (cases) was significantly lower (t(28) = −4.757, p = 0.000) than that of non-cases (3.90 ± 3.14 for the cases vs. 7.58 ± 4.58 for the non-cases). As for the HADS depression, equal variances were assumed according to the Levene’s test (F = 2.79, p = 0.097). The mean depression score on the HADS for patients who disavowed needs, ranging from 1 to 8, was also significantly lower (t(28) = −4.406, p = 0.001) than the mean depression score of the non-cases, ranging from 0 to 18 (4.35 ± 2.37 vs. 6.92 ± 3.49).

The mean score on the CAT for patients who disavowed needs (cases) was 15.6 ± 6.44 while the corresponding score for non-cases was 24.25 ± 7.10. Assumption of normality was met for the CAT score, as well as the assumption of equal variances (F = 1.31, p = 0.255). The difference in mean scores was statistically significant (t(24) = −5.645, p = 0.000).

Sixteen patients (80%) who disavowed needs reported not taking antibiotics for an exacerbation in the last month, whilst this was the case for 104 patients (57%) of the non-cases reported. However, the difference was not statistically significant (chi-square(2) = 6.221, p = 0.05).

The normality assumption and the assumption of equal variances were met for the CRQ score (F = 0.48, p = 0.49). For the patients who disavowed needs, scores for mastery on the CRQ had a mean value equal to 5.95 ± 1.19, while for non-cases, the mean value was significantly lower (t(26)= 5.845, p = 0.000) (4.34 ± 1).

Twelve of the twenty-one (57.1%) patients who disavowed needs (cases) had had contact with their GP in the past three months, while 20 cases reported that they wanted to have more contact with their GP; one did not answer. In the non-cases, 71.1% (150/214) had had contact with their GP in the past three months, however only 2.8% (6) reported that they wanted more contact with their GP. Neither the difference in contacts with their GP in the past three months, nor the need for more contact with the GP, was statistically significant between the cases (chi-square(1)=1.763, p = 0.184 and the non-cases (chi-square(1)=0.607, p = 0.436.

##### The effect of risk factors in relation to HCP involvement

First, we examined the effects of the patient’s age on having HCP involvement for the cases and the non-cases. The relationship between age and having HCP involvement was significant for the non-cases (p = 0.019), but not significant for patients who disavowed needs (p = 0.375). For the non-cases, the negative coefficient (b = −0.046) indicates that the older the patient the lower the likelihood of HCP involvement and more precisely, considering the exponentiation of this coefficient, for each year of patient’s life the likelihood of HCP involvement decreases slightly by 4.5% (Table 4).

**Table 4.** Logistic regression models for the effect of risk factors on the involvement of HCP (identification by patient of an HCP who had provided support in the last three months).

Group	Risk factor and constant	B	S.E.	Wald	df	Sig.	Exp(B)
Disavowal of need	Patient age	−0.055	0.062	0.785	1	0.375	0.947
	Constant	5.046	4.740	1.133	1	0.287	155.393
Absence of disavowal of need	Patient age	−0.046	0.019	5.476	1	0.019*	0.955
	Constant	4.805	1.448	11.004	1	0.001**	122.106

Disavowal of need	HADS anxiety	−0.142	0.162	0.766	1	0.382	.868
	Constant	1.436	0.868	2.740	1	0.098	4.206
Absence of disavowal of need	HADS anxiety	0.094	0.044	4.498	1	0.034*	1.098
	Constant	0.877	0.340	6.645	1	0.010*	2.404

Disavowal of need	HADS depression	−0.040	0.212	0.036	1	0.849	0.960
	Constant	1.024	1.060	0.934	1	0.334	2.786
Absence of disavowal of need	HADS depression	0.101	0.059	2.915	1	0.088	1.106
	Constant	0.867	0.413	4.399	1	0.036*	2.380

Disavowal of need	CAT score	0.086	0.097	0.779	1	0.377	1.090
	Constant	−0.172	1.463	0.014	1	0.907	0.842
Absence of disavowal of need	CAT score	0.071	0.028	6.575	1	0.010*	1.073
	Constant	−0.241	0.642	0.141	1	0.707	0.786

*p< 0.05, **p < 0.01, ***p < 0.001.

Regarding the effects of the HADS anxiety score on having HCP involvement, we found a significant effect for non-cases (p = 0.034), but this was not significant for patients who disavowed needs (p = 0.382). Particularly, for non-cases we found that for each unit of increase in HADS anxiety score, the likelihood of HCP involvement increases by 9.8% (Table 4). As for the relationship between the HADS depression score and the involvement of HCPs, we found no statistically significant effect for either group of patients (Table 4).

When we examined the effects of the CAT score on having HCP involvement, we found that the relationship between this score and HCP involvement was significant for non-cases (p = 0.010), but not significant for patients who disavowed needs (p = 0.377). For the non-cases, one unit increase in the CAT score increases the likelihood of HCP involvement by 7.3% (Table 4).

### Stage 2: Focus groups with healthcare practitioners in primary care

Key findings are summarised below in two sections: i) current experiences and challenges of working with ‘I’m Fine’ patients and ii) improving practice with ‘I’m Fine’ patients.

#### Current experiences and challenges of working with ‘I’m fine’ patients

HCPs described recognising the profile of patients who disavowed their medical needs. They recounted how they saw patients with COPD who were visibly breathless, but who would describe themselves as feeling “fine” and under-report their symptoms. They also noted how these patients would justify their position by dismissing their symptoms or comparing themselves to others who were worse off.

Some HCPs linked patient disavowal of needs with elements of non-compliance to recommended symptom management:

… they deny [experiencing breathlessness], or even if they admit it, they don’t want to take the inhalers. That’s the biggest thing. They don’t want to take the inhalers, ‘I’m alright,’ and that’s all you get. [HCP 1 FG2]

Examples were also given of patients choosing not to liaise with HCPs when they were experiencing a severe exacerbation of their symptoms, rejecting offers of support available via the surgery, or avoiding contact with HCPs over a long period of time while their condition deteriorated.

HCPs accounted for patient disavowal of support needs related to COPD in terms of difficulties accepting a deterioration in health, fear of the future, and guilt around smoking. Some HCPs also described disavowal of needs as a kind of coping strategy, and one that could sometimes be effective.

Discussion in the focus groups also identified specific challenges HCPs face in terms of engaging patients who disavow their needs:

I quite often think when they do say ‘I’m fine’ I'm thinking ‘do I need to say it's not fine or is there something we can do’, or do we do the ‘I’m fine ’because let's just leave them alone because there's nothing else we can offer? [HCP 3 FG1]

HCPs questioned whether they should accept patient choice and how to raise questions with patients sensitively, particularly where there was perceived limited scope for relieving symptoms.

#### Improving practice with ‘I’m fine’ patients

Some HCPs recounted how, in practice, better engagement frequently arose only in response to the patient experiencing a crisis or hospital admission. However, several outlined strategies that they or their service had developed to improve engagement and compliance. These included: 1) presenting information about symptom severity to patients using a visual format; 2) adopting a system of recall for patients who did not attend appointments and reviews; and 3) giving a strong message to patients that the door was always open if they needed to see someone. Suggestions for ways that engagement could be improved included more media campaigns about COPD, longer consultations and asking patients to reflect on, and write down issues that were important to them prior to attending an appointment.

## Discussion

Using criteria from studies of disavowal of needs in mental health, we identified 21 interview transcripts from a sample of 235 patients with advanced COPD which contained substantial discursive markers of i) a participant’s implicit recognition of needs combined with ii) explicit disavowal of needs. Further qualitative comparison of these interview transcripts (cases) with a random selection of other transcripts (non-cases) revealed two further characteristics. First was non-compliance with symptom management strategies. Patients disavowing their needs appeared more likely to describe a wish to manage without medical help and to regard their medicines as futile. These findings align with those of Caspers and colleagues who found reduced engagement with supportive services among patients in a substance-use clinic who disavowed needs during a clinical interview.^11^ A second finding was that participants in our sample who disavowed their needs seemed to avoid thinking about their future or future care, including avoiding discussing this with friends and family. Neither treatment non-compliance nor avoidance of planning for future care are intrinsically implied by disavowal of needs. However, they appear as intelligible characteristics for patients who adopt a strategy of directing attention away from their health and care needs. These findings suggest that the disavowal of needs in a clinical research context may be related to a desire to appear not to require medical advice or medicines, and a disinclination to think about or discuss future needs.

The 21 participants identified as disavowing their needs in interviews were compared to the rest of the sample on a number of measures. On widely-used, standardised self-report measures, these participants reported fewer mental health needs. This was in line with existing literature on disavowal of needs in the context of mental health. However, we found additionally that these patients reported fewer physical health needs, and higher scores for feelings of mastery over their chronic condition. This is a novel finding in relation to the literature on disavowal of medical needs. One clear possibility is that these participants simply were mentally and physically less unwell and/or have fewer needs than the rest of the sample. However, two findings are somewhat incompatible with this conclusion. One finding is that all of these patients stated that they wished to have more contact with their GP, despite lower rates of GP contact than the rest of the sample. This was in contrast to only six of the remaining 235 patients in the sample who said that they wished to have more contact with their GP. This suggests some reluctance among cases to make full use of primary care services, combined with a desire for more primary care support. A second finding incongruent with the idea that these patients are accurately reporting their lack of need came from the series of logistic regressions, which showed that the usual link between risk factors and greater HCP involvement in the rest of the sample was absent for these patients. This suggests that, as in the mental health literature, disavowal of need in the interviews in our study of COPD may indicate a psychological process (a coping strategy) that curtails the use of services when they are needed.

Focus groups with primary care HCPs indicated that patients who disavow their needs were a familiar profile to them. They also mentioned concerns about treatment non-compliance in these patients, in agreement with our qualitative interview analysis. More broadly the findings also suggest that patient disavowal of need can be understood as a performative action indicating that the activating conditions, or triggers, familiar to GPs are not met for certain kinds of institutional response. HCPs in this study reported experiencing dilemmas about how best to engage patients, or discuss needs that a patient seemed disinclined to recognise. Possible strategies identified that could be used in this situation included adopting a system of recall for patients who did not attend appointments and reviews and giving longer consultations for patients who might find it difficult to acknowledge their difficulties. They also suggested asking patients to write down issues that were important to them, prior to attending an appointment. It may be that patients with COPD who have difficulty expressing their support needs would benefit from structured forms of help with this.^23,24^

## Strengths and limitations

A key strength of this study was the use of multiple sources of data, from a large population-based sample of people with advanced COPD, enabling us to explore the ‘I’m Fine’ patient group from a number of perspectives. In addition, our study has several limitations. As secondary analysis of cohort data, the measures we drew upon were not chosen specifically to address our research questions. So for instance disavowal of need was measured through an application of an existing measure from the mental health literature to interviews conducted for another purpose, rather than on the basis of specifically-conducted interviews. This may have reduced the number of cases of disavowal we could identify. It also reduces comparability with the findings of studies of disavowal of mental health needs, since the measure was not quite the same. It also limited our ability to probe for relevant features of the lives of our participants, such as the dynamics that develop with family and health care providers that stem from individuals’ acknowledgment or disavowal of support needs. The findings from our focus groups suggest that this would be an important area for further study. Another limitation was our use of random selection of non-cases for comparison rather than case-matching. Since this was exploratory work, we did not have sufficient certainty about the expected covariates for disavowal or acknowledgement of needs, and so lacked a basis for criteria against which cases could be matched. However, this will have reduced the acuity of the comparison. In addition, findings from the logistic regressions should be taken prudently due to the sample size of the case and the control groups. However, it should also be noted that, despite the small size of the case sample in comparison to the non-case population, the rule of thumb of 10 events per variable is still met for the non-cases, supporting the overall acceptability of these results.^25^ Finally, whilst our findings suggest that disavowal of needs may have relevance to both mental and physical health, we cannot know whether this patient group is accurately reporting their reduced level of need. Further research is required with clinician, patient-reported and carer-reported measures to explore this further.

## Conclusion

We set out to study how people with advanced COPD disavow their support needs and how this influences their care. We found 21 interview transcripts from a sample of 235 patients with advanced COPD that contained substantial discursive markers of implicit recognition of needs combined with explicit disavowal of needs. These 21 patients appeared to have several notable characteristics such as apparent non-compliance with symptom management strategies, a wish for more contact with their GP despite lower rates of GP contact, and no relationship between risk factors and extent of health care professional involvement.

The findings of this study have implications both for understanding the complexities of identifying patient needs in COPD care generally, and for primary care. There seemed a real desire among patients who disavowed need in interview to have more contact with their GP. But lower attendance at the GP than the rest of the sample suggests that they may have reservations or difficulties in bringing problems to the GP themselves, or are using it as a self-management and coping strategy. Though far from conclusive, there are some indications in our data that these patients are underreporting their need. If so, this may have relevance to how clinicians interpret reports of exacerbations or scores on self-report measures of chronic symptoms such as the CAT. Our findings also suggest that treatment adherence, or perhaps reliance on or trust in healthcare services in general, may be a challenge for this group of patients. Future research may wish to explore further the specific support needs of these patients in contrast to non-cases, the experiences of secondary care services with these patients and also how these patients can best be supported to engage with services and make full use of their medication.
